# Genetic basis of Charcot-Marie-Tooth disease in Pakistani consanguineous families

**DOI:** 10.3389/fneur.2026.1920139

**Published:** 2026-09-10

**Authors:** Zafar Ali, Muhammad Jameel, Joakim Klar, Muhammad Suleman, Uswah Batool, Hammad Yousaf, Farhan Ali, Ambrin Fatima, Shahid Mahmood Baig, Niklas Dahl, Mathais Toft, Uzma Abdullah, Zafar Iqbal

**Affiliations:** 1 Department of Biotechnology, Genetics, Forensic and Microbiology, University of Swat, Charbagh, Swat, Pakistan; 2 Centre of Oncological Research in Surgery, The Aga Khan University, Karachi, Pakistan; 3 Department of Immunology, Genetics and Pathology, Science for Life Laboratory, Uppsala University, Uppsala, Sweden; 4 Biomedical Research Center (BRC), QU Health, Qatar University, Doha, Qatar; 5 University Institute of Biochemistry and Biotechnology, Pir Mehr Ali Shah Arid Agriculture University, Rawalpindi, Pakistan; 6 Department of Biological and Biomedical Sciences, The Aga Khan University, Karachi, Pakistan; 7 Frontier Corps Teaching Hospital Hayatabad Phase-6, Peshawar, Pakistan; 8 Faculty of Life Sciences, Health Services Academy, Islamabad, Pakistan; 9 Department of Neurology, Oslo University Hospital, Oslo, Norway; 10 Institute of Clinical Medicine, University of Oslo, Oslo, Norway

**Keywords:** Charcot-Marie-Tooth disease, *GDAP1*, Pakistani, recessive, *SBF2*

## Abstract

Charcot-Marie-Tooth (CMT) is a group of inherited neuromuscular disorders with diverse clinical features such as muscle weakness and atrophy of the distal regions, foot deformities, sensory loss and decreased or absent reflexes. With the diverse inheritance patterns including dominant, recessive, and X-linked, it exhibits significant genetic heterogeneity often overlapping with other neurological disorders. Whole exome sequencing (WES) has emerged as favorable diagnostic tool for such challenging disease entities. We recruited five Pakistani families that consisted of 14 affected individuals exhibiting representative symptoms of CMT such as severe muscle weakness of the lower limbs, muscles atrophy, claw hands and feet deformities. WES data analysis revealed two novel and two already reported homozygous missense variants in *GDAP1* (NM_018972) c.1A > G (p. Met1Val), c.683A > G (p. Glu228Gly), c.734C > T (p. Thr245Ile) and c.811G > A (p. Gly271Arg) in families A–D, respectively. While a novel homozygous *SBF2* variant c.2746G > C, p. (Gly916Arg) was identified in family E. Sanger sequencing confirm the segregation of all candidate variants in their respective pedigrees. Moreover, molecular modeling supports the destabilizing effect of these variants on final protein product of both *GDAP1* and *SBF2* by inducing modifications in overall conformation of the protein. The present study expands the mutation spectrum of the *GDAP1* and *SBF2* genes, with possible implications for improved understanding of disease pathophysiology. Furthermore, the current study highlights the importance of NGS, particularly WES, in achieving rapid and precise molecular genetic diagnosis for clinical purposes, especially in neuromuscular disorders of heterogeneous nature.

## Introduction

Charcot-Marie-Tooth (CMT) disease is the most common form of inherited neuromuscular disorder. The global prevalence of CMT is one in 2,500 (1). It exhibits substantial clinical heterogeneity, though main features include muscle weakness and atrophy of the distal regions, initially of the lower limbs and spreading to the upper limbs, foot deformities, and sensory loss and absent or decreased reflexes (1–3). Clinical sub classification of CMT is assigned on the basis of electrophysiological findings and these include demyelinating, axonal and an intermediate type (4, 5). CMTs are observed in all possible forms of inheritance modes, with number of reported genes exceeding 140 (6–9). The association of several genes with CMT disease indicates the involvement of multiple cellular pathways. Gene variants affecting the synthesis of myelin sheath, intracellular transport, gap junction communication, mitochondrial biology, and transcriptional regulation all disrupt peripheral nerve structure or function. These diverse pathogenic mechanisms ultimately converge on progressive axonal loss and peripheral neuropathy phenotypes (1, 10). Individuals with CMT have a compromised physical, social, and emotional quality of life, the physical aspect being the most affected (1). Treatment for CMT is primarily supportive and aims to improve the patient’s functional abilities and quality of life. Rehabilitative care through physiotherapy and occupational therapy helps to improve balance, mobility and strengthen the muscles (11). Early diagnosis of the disease can allow for appropriate management and care. Despite continuous ongoing research and identification of almost 140 genes to be the cause of CMT, still 40% of the CMT cases remain genetically undiagnosed (12, 13).

Herein we have analyzed five consanguineous Pakistani families that comprise of 14 affected individuals segregating severe muscle weakness of the lower limbs, muscles atrophy, claw hands and feet deformities. WES identified four missense variants in *GDAP1* and a missense variant in *SBF2*, each one in the homozygous form. The findings from this study expand the genetic spectrum of this complex neuromuscular disorder.

## Materials and methods

### Study subjects & clinical investigations

We recruited five inbred families of Pakistani origin. Affected individuals in all five families segregating muscle weaknesses at the distal regions of both lower and upper limbs, muscle atrophy and diminished sensation in distal region of the limbs. Written informed consents were taken from all participants and/or their legal custodians. A total of 31 individuals including 14 affected and 17 unaffected individuals were sampled. Multiple members of the families were interviewed for clinical history of the disease and pedigree drawing. The patients were physically examined for phenotypic abnormalities and subjected to NCS and EMG studies by expert neurologists.

### Genetic analysis

Blood samples were obtained from all affected and phenotypically normal individuals of all five families. The phenol-chloroform method was used to isolate the DNA from all blood samples (14). We opted for whole exome sequencing due to the complex nature of the disease both clinically and genetically.

WES was conducted on DNA, from affected individuals of family A, B and E (Family A-IV:2 & V:3; Family B-IV:1 & IV:4; Family E-IV:4) using the Ion Proton System (Life Technologies, Carlsbad, CA, USA) as described previously (15).

The reads were aligned to the human reference sequence (hg19 assembly) and variants were detected using v2.1 of the LifeScope Software (Life Technologies). SNPs and indel data were stored in an in-house exome database together with variant annotation information obtained from ANNOVAR and dbSNP135 (16). Potentially damaging variants were identified using Custom R scripts (17). While WES on DNA from affected individuals (Family C–IV:1; Family D–IV:3) from Family C and D was performed as previously described (18). The priority was given to homozygous and compound heterozygous variants, due to a suspected recessive mode of inheritance in all five families.

Segregation analysis was performed by bidirectional Sanger sequencing (Applied Biosystems Big Dye Terminator v3.1 Cycle Sequencing Kit, Applied Biosystems, Carlsbad, CA, USA; Life Technologies) on a 3730xl DNA Analyzer (Applied Biosystems, Life Technologies). Sanger sequencing results were analyzed using Sequencher software (Gene Codes Corporation, Ann Arbor, MI, USA).

### Molecular modeling and structural validation

The amino acid sequence of both *GDAP1* and *SBF2* were utilized as input for generating the 3D structure through application of AlphaFold 2.0. This innovative protein folding prediction system is developed by DeepMind, a research lab under Alphabet Inc., and employs deep learning methods to predict the three-dimensional structure of proteins (19). To enhance the reliability of the results, additional validation procedures were conducted using ProSA-web and PROCHECK. These validations encompassed the examination of the Ramachandran plot to verify the appropriate distribution of amino acid residues and bond angles (20, 21). The identified variants in both genes were subjected to the mCSM server^1^ to determine the effect of candidate variants on protein stability by using graph-based signature. By analyzing the ΔΔG (difference in free energy) values for each candidate variant, the mCSM gained valuable insights into how these variants affected the stability of the proteins under investigation (22). The Chimera software was employed to incorporate the candidate highly destabilizing variants into the wild type structure of the *GDAP1* and *SBF2* protein (23). After conducting stability assessments, we utilized the PyMOL software to compare the structural variances between the wild type (WT) and its mutant forms of both *GDAP1* and *SBF2*. This was accomplished by superimposing each mutant with the WT *GDAP1* and *SBF2* structures and then computing the RMSD (root mean square deviation) value. This approach enabled us to measure and gain insights into the degree of structural alterations introduced by both *GDAP1* and *SBF2* variants in comparison to the wild-type protein configuration.

## Results

### Clinical information

**Figure 1:**
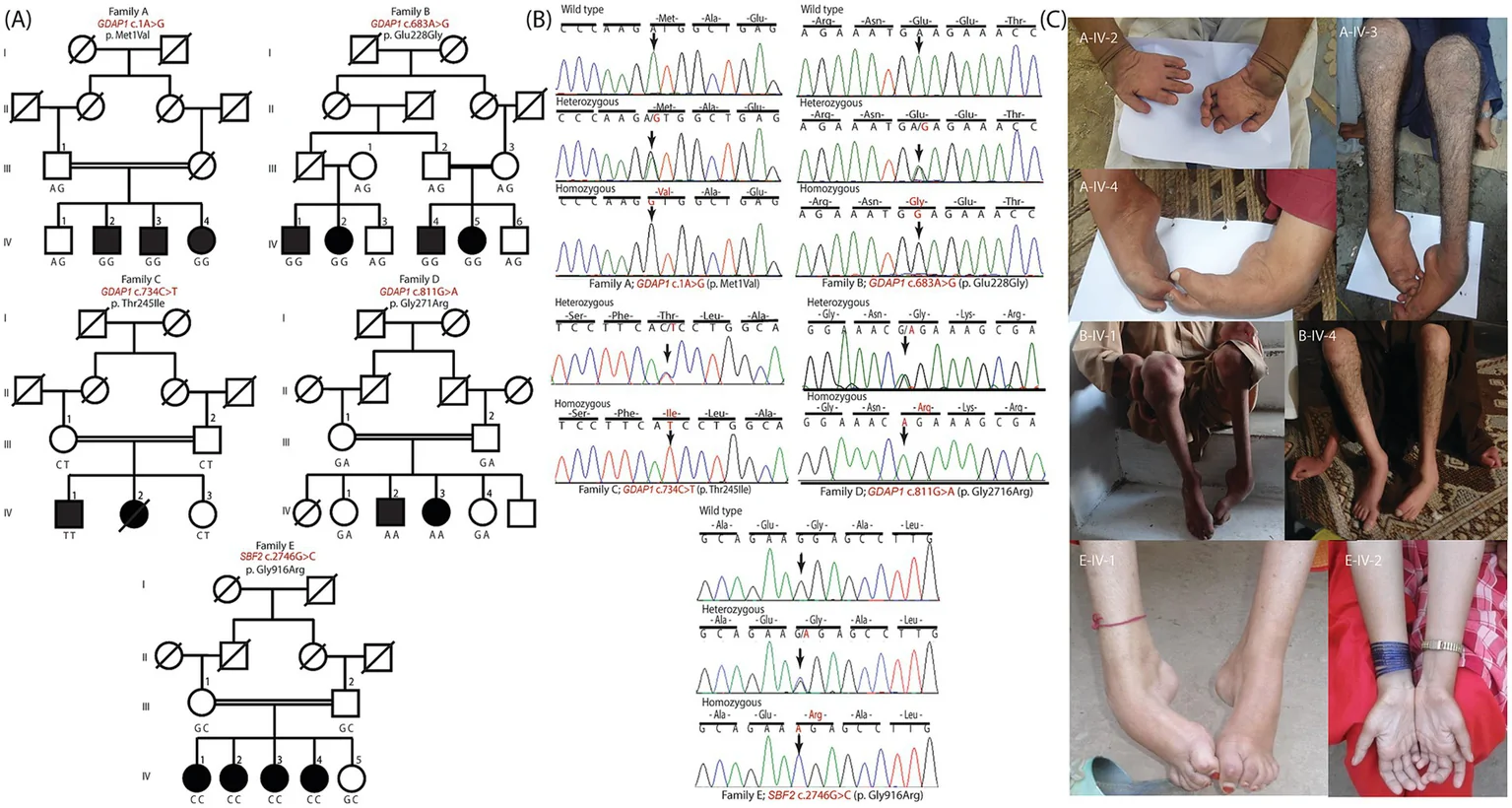
Pedigrees of the five families segregating *GDAP1* and *SBF2* variants. **(A)** Pedigrees of Families A–E. Filled symbols represent affected individuals. Identified variants are written in red on top of pedigrees. Genotypes are shown for each tested individual below the symbols. **(B)** Sequence chromatogram of candidate region of *GDAP1* and *SBF2* gene containing the variant. **(C)** Clinical photographs of the individuals carrying homozygous *GDAP1* and *SBF2* variants.

Family A (Figure 1A) was sampled from district Swat, Khyber Pakhtunkhwa, Pakistan. The family has three affected siblings (two males and one female) born to a healthy consanguineous couple and aged 27, 24 and 22 years at the time of examination. Their developmental milestones were slightly delayed, i.e., started sitting at 8 months, walking without support at 15–16 months of age. The initial symptoms, including muscle weakness and easy fatigability, manifested at approximately 36 months of age. Additionally, they started losing their hand grip and developed claw hand and foot deformities later. They also suffered from severe muscle atrophy which is evident from the images (Figure 1C). The disease worsened over time and by the end of the second decade of life they were completely wheelchair dependent. Furthermore, their intellect, speech and vision were normal and there was no other abnormality reported.

Family B (Figure 1A) also originated from district Swat of Khyber Pakhtunkhwa Province, Pakistan. This extended family had 4 affected individuals born to two consanguineous parents. They had unremarkable gestation and delivery and achieved developmental milestones a little late such as their first roll over was between 5 and 6.5 months of age, independent walk at 15–16 months except individual IV:5, who never walked. The parents initially observed complaints of muscle weaknesses between 4 and 6 years of age and noticed frequent falls of affected individuals on daily basis. There was reduced sensation in the limbs, more pronounced in the lower limbs compared to the upper limbs. This condition was progressive, leading them to be wheel-chair dependent in their late adolescence. Moreover, their intellect was intact, and there was no speech or visual impairment.

Family C (Figure 1A) also has a Pakistani origin. One affected individual born to a healthy couple who are first cousins is alive. Initially he had difficulties with walking and abnormal gait. Moreover, he also had impaired sit-to-stand transition and stair climbing limitation. The parents reported an unremarkable neonatal history. According to them, he achieved all motor and developmental milestones until the age of 1–1.5 years. After that progressive walking abnormalities developed. Initially only the left side was affected, but later it was bilateral and affected upper limbs as well. At the time of examination, he had high stepping and waddling gait. Atrophy of the muscles was also prominent. Power was overall decreased, which was more prominent in distal muscle group of hands and feet. No tongue fasciculations were found. Gowers and Romberg’s sign were positive, cerebellar signs such as nystagmus and past pointing were absent. All cranial nerve functions were preserved. Superficial abdominal reflex was also intact. He was also able to appreciate touch and pain sensation, but position sense was lost. Moreover, there was no visual impairment.

Family D (Figure 1A) originated from upper Punjab, Pakistan and two family members presented with an inherited neuromuscular disorder. The clinical information of the affected individuals is summarized in Table 1. Briefly, they showed normal early developmental milestones followed by late childhood-onset motor regression, frequent falls, and lower limb joint locking, without any intellectual disability. The inheritance pattern is consistent with autosomal recessive transmission. Patient IV:4 is a 9.5-year-old female with a height of 119 cm (−3 SD) and head circumference of 51 cm (−2.5SD). She was born via normal delivery and achieved early motor milestones appropriately: she first rolled over at 6 months of age and began walking without support at 1.25 years (15 months). Her social smile was reported as good. Following a period of normal development, she experienced motor regression starting after 9 years of age. She frequently gets foot or ankle locking, leading to falls approximately 1–2 times per day. She gets tired easily with activity. Importantly, there is no intellectual disability, and cognitive function remains intact. Patient IV:5 is a 12-year-old male with a height of 127 cm (−3 SD) and head circumference of 52 cm (−2.5 SD). He was born via normal delivery and achieved early motor milestones like his affected sibling: first rolling at 6 months and walking independently at 1.25 years (15 months), with a good social smile. Regression of motor skills also began after 9 years of age. He experiences foot or ankle locks and falls 1–2 times per day, and he gets tired easily. There is no intellectual disability. His past medical history is notable for broken teeth due to an accident and episodes of fainting lasting 2–4 s during childhood.

**Table 1:** Genetic and clinical findings of patients segregating GDAP1 and SBF2 variants.

Clinical parameter	Family A			Family B				Family C	Family D		Family E			
	IV-2	IV-3	IV-4	IV-1	IV-2	IV-4	IV-5	IV-1	IV:2	IV:3	IV-1	IV-2	IV-3	IV-4
Age at examination (years)	27	24	22	26	23	28	23	7 years	9.5	12	19	17	14	12
Sex	Male	Male	Female	Male	Female	Male	Female	Male	Female	Male	Female	Female	Female	Female
Genotype	*GDAP1 (NM_018972.2):* c.1A > G	*GDAP1* c.1A > G	*GDAP1* c.1A > G	*GDAP1* c.683A > G	*GDAP1* c.683A > G	*GDAP1* c.683A > G	*GDAP1* c.683A > G	*GDAP1* c.734C > T	*GDAP1* c.811G > A	*GDAP1* c.811G > A	*SBF2* (NM_030962.4): c.2746G > C	*SBF2* c.2746G > C	*SBF2* c.2746G > C	*SBF2* c.2746G > C
Status	Reported (PubMed: 33,179,230)	Reported (PubMed: 33,179,230)	Reported (PubMed: 33,179,230)	Novel	Novel	Novel	Novel	Novel	Reported (PubMed: 14561495)	Reported (PubMed: 14561495)	Novel	Novel	Novel	Novel
ACMG classification	Pathogenic	Pathogenic	Pathogenic	VUS	VUS	VUS	VUS	Likely Pathogenic	Likely Pathogenic	Likely Pathogenic	VUS	VUS	VUS	VUS
ACMG criteria	PVS1. PP5. PM2	PVS1. PP5. PM2	PVS1. PP5. PM2	PM1, PM2	PM1, PM2	PM1, PM2	PM1, PM2	PM1, PP3, PM2, PP1	PM1, PM2, PP3, PP5	PM1, PM2, PP3, PP5	PP3, PM2	PP3, PM2	PP3, PM2	PP3, PM2
gnomAD exomes v4.1	0.00000138	0.00000138	0.00000138	Absent	Absent	Absent	Absent	Absent	0.0000328	0.0000328	Absent	Absent	Absent	Absent
Clinvar submission	RCV005047425, RCV003105618	RCV005047425, RCV003105618	RCV005047425, RCV003105618	Absent	Absent	Absent	Absent	Absent	6 submissions	6 submissions	Absent	Absent	Absent	Absent
Clinvar classification	Pathogenic	Pathogenic	Pathogenic	n/a	n/a	n/a	n/a	n/a	Conflicting (P:2; LP:1; VUS:3)	Conflicting (P:2; LP:1; VUS:3)	n/a	n/a	n/a	n/a
Birth history	Normal delivery	Normal delivery	Normal delivery	Normal delivery	Normal delivery	Normal delivery	Normal delivery	Normal delivery	Normal delivery	Normal delivery	Normal delivery	Normal delivery	Normal delivery	Normal delivery
CMTNS	25	27	26	33	31	34	32	21	15	12	28	27	28	26
First rolling	5 months	5 months	6 months	6.5 months	6 months	5 months	6 months	5 months	6 months	6 months	5.5 months	6 months	6 months	5 months
Walking without support (months)	16	15	15	15	15	16	abasia	13	15	15	14	15	15	14
Social smile	Good	Good	Good	Good	Good	Good	Good	Good	Good	Good	Good	Good	Good	Good
Age of regression onset	After 3 years	After 3 years	After 3 years	After 6 years	After 4 years	After 5 years	abasia	1.6 years	After 9 years	After 9 years	After 6.5 years	After 7 years	After 5 years	After 5 years
Foot/ankle locks	Yes	Yes	Yes	Yes	Yes	Yes	Yes	Yes	Yes	Yes	Yes	Yes	Yes	Yes
Falls per day	WD	WD	WD	WD	WD	WD	WD	1–2 times	1–2 times	1–2 times	assisted ambulation	assisted ambulation	assisted ambulation	assisted ambulation
Easy fatigability	Yes	Yes	Yes	Yes	Yes	Yes	Yes	Yes	Yes	Yes	Yes	Yes	Yes	Yes
Intellectual disability	No	No	No	No	No	No	No	No	No	No	No	No	No	No
Other medical history	None reported	None reported	None reported	Aggressive	Aggressive	Aggressive	Aggressive	None reported	None reported	Fainting episodes (2–4 s) in childhood	None reported	None reported	None reported	None reported

VUS: Variant of Uncertain Significance, PM: Pathogenic Moderate, PVS: Pathogenic Very Strong, PP: Pathogenic Supporting, CMTNS: CMT Neuropathy Score, WD: wheelchair-dependent.

Family E (Figure 1A) also has a Pakistani origin with four affected females born to first-degree consanguineous healthy parents. All the patients achieved developmental milestones normally. They had difficulties with walking and grasping things through hands initially. Patients manifested clinical symptoms at approximately 12 years of age with progressive weakness and numbness of hands and feet. Patients were unable to walk without support at the time of investigations. Besides abnormal ambulation, prominent foot flexion deformities, pes cavus, hammer toes and corkscrew deformity (inverted Champagne bottle atrophy) were also observed among the patients. There were no reflexes and they also showed stocking-glove pattern of sensory loss up to knees and elbows. Position sense was found impaired in all affected individuals. Their cognitive functions, hearing and visual capacities were intact.

Nerve conduction studies (NCS) and electromyography (EMG) of affected individuals IV-3 and IV-1 from family A and family E, respectively, were performed (Figure 1). While no individual from family B, C and D was available for NCS and EMG. NCS and EMG were performed using surface electrode and concentric needle electrode, respectively. In Family A, the findings were consistent with sensorimotor neuropathy with significant axonal involvement. No CMAP responses could be elicited in upper or lower extremities, therefore conduction velocities could not be calculated in all examined nerves, while EMG demonstrated neurogenic changes. The anterior tibialis and vastus medialis muscles showed no voluntary or spontaneous activity. In contrast, the deltoid, biceps, and trapezius muscles exhibited large, polyphasic motor unit action potentials (MUAPs) with a discrete interference pattern, without evidence of fibrillation potentials or positive sharp waves. In Family E, nerve conduction studies revealed conduction velocities of 6.7 m/s and 13.8 m/s in the right median and ulnar nerves, respectively. Overall, the electrophysiological findings are indicative of a mixed type sensorimotor polyneuropathy, predominantly demyelinating with secondary axonal involvement, more pronounced in the lower limbs.

### Genetic analysis

Data obtained from WES were analyzed both for homozygous and compound heterozygous variants due to the recessive mode of inheritance. The variants were further stratified based on disease relevance, presence in population databases, or the predicted impact of pathogenicity using *in silico* tools. The analysis revealed four homozygous missense variants in *GDAP1* [(NM_018972.2): c.1A > G, (p. Met1Val); c.683A > G, (p. Glu228Gly); c.734C > T, (p. Thr245Ile); c.811G > A, (p. Gly271Arg)] in family A, B, C and D, respectively. While a homozygous variant in the *SBF2* gene (NM_030962.3: c.2746G > C, p. Gly916Arg) was identified in family E. Due to the absence of any other strong candidate gene, *GDAP1* and *SBF2* were considered to be the most likely disease causing genes based on their previous association with Charcot Marie Tooth disease, a disorder having comparable clinical features as we have witnessed in our families. Bidirectional Sanger sequencing confirmed the segregation of the candidate variants in their respective pedigrees [Figure 1A (family A–E) & B]. The phenotypically healthy individuals of both families’ including parents and siblings of affected individuals were all heterozygous carriers for the wild type allele. The *GDAP1* variant (c.1A > G) in family A results in loss of the primary start codon (p. Met1Val) while variant c.683A > G in family B leads to a substitution of glutamic acid by glycine at residue number 228 (p. Glu228Gly). The *GDAP1* variant c.734C > T in family C result in amino acid change at position 245 (p. Thr245Ile) where a hydrophilic amino acid “Threonine” is replaced by a hydrophobic amino acid Isoleucine. Furthermore, the *GDAP1* variant (c.811G > A) in family D and *SBF2* variant (c.2746G > C) in family E changes Glycine, a non-polar amino acid to Arginine, a positively charged amino acids at position 271 (p. Gly271Arg) and 916 (p. Gly916Arg) respectively. The identified variants are highly conserved and are predicted to be ‘deleterious’ by various bioinformatics prediction tools. The four *GDAP1* variants were in exon 1, 5, and 6 in families A, B, C, and D respectively, while the *SBF2* variant in family E was in exon 22.

### Bioinformatics analysis

**Figure 2:**
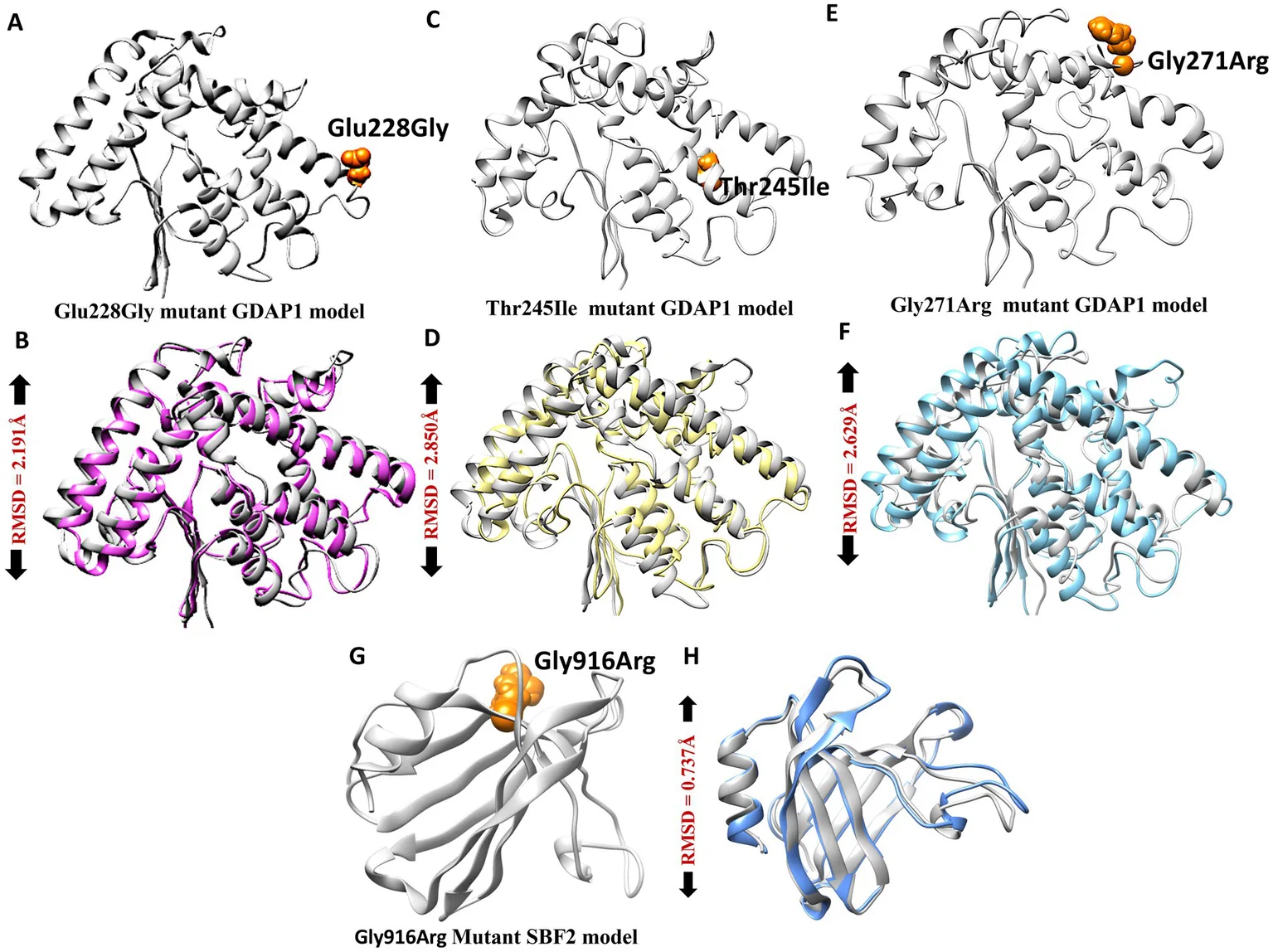
Mutants modeling and superimposition. **(A)** Represents the Glu228Gly mutant model of GDAP1. **(B)** Showing deviation of Glu228Gly mutant as RMSD. **(C)** Represents the Thr245Ile mutant model of GDAP1. **(D)** Showing deviation of Thr245Ile mutant as RMSD. **(E)** Represents the Gly271Arg mutant model of GDAP1. **(F)** Showing deviation of Gly271Arg mutant as RMSD. **(G)** Represents the Gly916Arg mutant model of SBF2. **(H)** Showing deviation of Gly916Arg mutant as RMSD.

The three-dimensional (3D) arrangement of a protein is crucial for understanding its function and interactions within the body. In case of *GDAP1*, its 3D structure was modeled using AlphaFold 2.0 (19), as shown in Supplementary Figure 1A. To validate the accuracy of the predicted *GDAP1* model, we conducted Ramachandran and ProSA-web analyses. The Ramachandran analysis revealed that 85.4% of amino acid residues occupied the most favorable regions, while 14.2% were in permissible areas, and only 0.4% were in disallowed regions, as depicted in Supplementary Figure 1B. Furthermore, the ProSA-web analysis generated a *z* score of −7.46 for the predicted *GDAP1* protein structure (Supplementary Figure 1C), a value consistent with the expected range for normal protein structures of comparable size (24). The candidate variants (Met1Val, Glu228Gly, Thr245Ile and Gly271Arg) underwent analysis using the mCSM server, which indicated that the prioritized variants have a significant destabilizing effect. The calculated ∆∆G values were −0.36 kcal/mol for Met1Val, −0.804 kcal/mol for Glu228Gly, −0.185 kcal/mol for Thr245Ile and −0.29 kcal/mol for Gly271Arg, suggesting high destabilization caused by these variants. Additionally, the candidate *GDAP1* destabilizing variants (Met1Val, Glu228Gly, Thr245Ile and Gly271Arg) were modeled into the wild type *GDAP1* structure using Chimera (Supplementary Figure 2B and Figures 2A,C,E).

To assess the structural difference between the wild-type *GDAP1* protein and the generated mutants, a superimposition of their respective structures was performed, followed by the calculation of root-mean-square deviation (RMSD) values. The obtained RMSD values pointed towards significant differences between the mutants and the wild-type protein. Specifically, the p. Met1Val, p. Glu228Gly, p. Thr245Ile, and p. Gly271Arg variants exhibited RMSD values of 2.225 Å, 2.19 Å, 2.850 Å, and 2.629 Å, respectively (Figures 2B,D,F; Supplementary Figure 2C). These observed variants were responsible for inducing modifications in both the secondary structure and overall conformation of the protein.

Similar analysis for *SBF2* variant (c.2746G > C, p. Gly916Arg) predicted excellent stereochemical quality of its Alpha fold generated protein model (Supplementary Figure 1D), with 92.1% of residues in the most favored regions, 7.9% in additionally allowed regions, and 0.0% in disallowed regions. The model was validated by using a Ramachandran plot (Supplementary Figure 1E). ProSA-web analysis produced a *z*-score of −4.11, which lies within the expected range for native proteins of similar size, confirming the reliability of the predicted model (Supplementary Figure 1F). The mCSM server predicted a ΔΔG value of −0.907 kcal/mol for the p. Gly916Arg, indicating a destabilizing effect on the *SBF2* protein. To further assess the impact of the variant on the structural integrity of the SBF2 protein, the variant was modeled within the wild-type protein structure (Figure 2G). Superimposition of the wild-type and mutant structures showed an RMSD of 0.737 Å (Figure 2H), suggesting noticeable conformational changes. Overall, the p. Gly916Arg variant destabilizes the SBF2 protein and induces structural perturbations that may affect its functional integrity.

## Discussion

We studied five consanguineous unrelated Pakistani families segregating a neuromuscular disorder, characterized by muscular weakness and atrophy more prominent and severe in upper limbs as compared to lower limbs, foot deformities and sensory loss at the distal regions. Patients initially complained of muscular weakness in the lower limbs, which later progressed to distal regions of the body involving the upper limbs. At this stage the patients were struggling to walk without support. There were prominent foot flexion deformities in the patients (pes cavus, hammer toes, pes equinovarus) and corkscrew deformities (inverted Champaign bottle atrophy).

Two novel and two known homozygous missense variants were identified in *GDAP1* in family A–D, while a novel homozygous *SBF2* variant was identified in family E. Sanger sequencing confirm the segregation of all candidate variants across their respective pedigrees. The *GDAP1:* c.1A > G (rs1474390668) (25) variant in family A is speculated to cause the loss of initiation codon of *GDAP1* protein which is essential for translation initiation, while the *GDAP1* variant (c.683A > G) in family B is a change of A to G at position 683. The protein features, confirmation or structure might be affected due to the substitution of highly conserved amino acid “glutamic acid” to “glycine” (p. Glu228Gly) and hence its localization. Family C segregated a novel *GDAP1* variant (c.734C > T), that results in substitution of a hydrophilic amino acid Threonine to Isoleucine which is hydrophobic (p. Thre245Ile) in nature. A hydrophobic amino acid tends to localize to the inner side of the protein, whereas hydrophilic ones are generally surface exposed; therefore, substitution of a hydrophilic residue with a hydrophobic one may disrupt protein folding which may cause significant structural modification in protein structure. Family D segregated a homozygous missense variant *GDAP1* c.811G > A, (p. Gly271Arg; rs775622226). This is the first report of this variant in homozygous form. The same variant have previously been identified in compound heterozygous state (with another variant *GDAP1* c.358C > T, p. Arg120Trp) in a female patient of Belgian origin, inherited from an unaffected mother and also observed in the heterozygous state in an unaffected sister (26). The affected siblings (IV:4 and IV:5) from Family C show notable phenotypic overlap with the previously reported patient carrying the same variant in compound heterozygosity with c.358C > T, (p. Arg120Trp) (26). In both families, the disease is characterized by progressive motor impairment predominantly affecting the lower limbs, resulting in gait difficulties, frequent falls, and loss of motor function over time. The absence of intellectual disability in our patients is also consistent with the previously reported phenotype, which was primarily limited to peripheral neuropathy without significant cognitive involvement.

The major difference between the two reports is disease severity and age of onset. The published patient developed symptoms at 3 years of age and progressed to wheelchair dependence by 20 years, whereas our patients had normal early motor development and remained ambulatory at 9.5 and 12 years of age despite the onset of motor regression after 9 years. However, both siblings already demonstrate evidence of disease progression, including recurrent falls, foot and ankle locking, easy fatigability, and declining motor function, suggesting that they may represent an earlier or milder manifestation within the same clinical spectrum. Variable expressivity and differences in disease severity are well recognized in inherited neuropathies, including Charcot-Marie-Tooth disease. Taken together, the occurrence of the same variant in unrelated affected individuals, the overlapping core phenotype of progressive motor neuropathy, and the segregation of the variant with disease in two affected siblings strongly support its pathogenic role. The milder presentation observed in our family likely expands the phenotypic spectrum associated with this variant rather than arguing against its clinical significance. Similarly, the *SBF2* variant (c.2746G > C) in family E changes Glycine, a non-polar amino acid to Arginine, a positively charged amino acids at position 916 (p. Gly916Arg). The candidate *SBF2* variant (c.2746G > C, p. Gly916Arg) destabilizes its protein by inducing structural perturbations that may affect its functional integrity as predicted by bioinformatics analysis.

Molecular modelling of the identified *GDAP1* variants predict their pathogenicity and revealed that all the variants, c.1A > G (p. Met1Val), c.683A > G (p. Glu228Gly), c.734C > T (p. Thre245Ile), and c.811G > A (p. Gly271Arg) are highly destabilizing, reflected by their negative ΔΔG values (−0.36, −0.804, −0.185 and −0.29 kcal/mol, respectively). Structural superimposition of mutant and wild-type *GDAP1* revealed significant deviations, with RMSD values of 2.22 Å for p. Met1Val, 2.19 Å for p. Glu228Gly, 2.850 Å for p. Thre245Ile and 2.629 Å for Gly271Arg suggesting that these variants introduce conformational alterations that could compromise the protein’s native stability and potentially interfere with its biological function. Such alterations are consistent with the previously reported effects of destabilizing mutations that perturb protein folding and structural integrity, often leading to impaired function or pathogenic consequences. Collectively, these findings indicate that the identified *GDAP1* variants may have deleterious effects on protein conformation and stability, offering molecular-level insights into their potential role in disease pathogenesis. Similarly, the *SBF2* variant was found in the highly conserved GRAM domain of the *SBF2* protein. Various *in silico* tools such as Mutation Taster and PolyPhen-2 predict it as deleterious. Similarly to *GDAP1*, the bioinformatics analysis of both *SBF2* wild and mutant type protein revealed a destabilizing effect of the *SBF2* variant (c.2746G > C; p. Gly916Arg) as evident from the ΔΔG value which is −0.907 kcal/mol after examining its structural effect using mCSM server. Moreover, the *SBF2* variant is most likely to introduce conformational changes in its final protein as shown by the superimposition of the wildtype and mutant structures followed by RMSD calculation having a deviation of 0.737 Å. Overall, computational analyses indicate the pathogenicity of the *SBF2* variant in family 3 by inducing alterations in both secondary structure and overall protein conformation, thus significantly destabilizes the SBF2 protein and induces structural abnormalities and modification that may affect its functional integrity.

*GDAP1* is mainly expressed in neurons and localizes to the outer membrane of mitochondria, where it plays a crucial role in the dynamics of mitochondrial networks (27, 28). *GDAP1* gene variants disturb the usual function of mitochondria such as disturbances in the fusion and fission processes of mitochondria, alterations in their distribution, hindered mitochondrial transmembrane potential, heightened levels of reactive oxygen species, diminished glutathione levels, and changes in mitochondrial energy production and leads to CMT (28–33). The typical mitochondrial function is vital for peripheral nerves due to their high energy demand of axons due to its long length (34). A *GDAP1* knockout mouse model has been reported to display a comparable phenotype to CMT characterized by peripheral neuropathy with the loss of motor neurons and unusual neuromuscular junctions (35), that is consistent with those observed in humans.

Previous reports of Pakistani families harbouring truncating, missense, and splice-site variants in *GDAP1* have described a relatively consistent clinical spectrum characterized by progressive distal weakness, fatigability, sensory impairment, muscle atrophy, foot deformities, curved fingers, diminished or absent reflexes and valgus ankles. However, the phenotypic severity has been variable depending on the underlying variant (loss of function vs. missense) (36–39). The affected individuals in our cohort carrying the novel and known missense variants exhibited a largely overlapping phenotype, further supporting the characteristic clinical presentation associated with *GDAP1*-related neuropathy in the Pakistani population. However, some degree of phenotypic variability was evident. For example, dysphonia, scoliosis and high-steppage gait with foot drop which has been reported in certain Pakistani patients with *GDAP1* variants (37–39) were absent in all the affected individuals from the families presented here. These findings demonstrate that the core clinical manifestations of *GDAP1*-associated neuropathy are largely consistent across Pakistani families, whereas differences in the presence and severity of specific features underscore the clinical heterogeneity observed among the affected individuals.

The *SBF2* gene is extensively expressed in almost all human tissues, with predominant expression in the nervous system. Expression analyses demonstrated that *SBF2* transcripts are detectable in multiple tissues, including the brain, spinal cord, and peripheral nerves, consistent with its established role in neuropathies. Subsequent studies and expression databases have shown that *SBF2* exhibits ubiquitous but variable expression, with relatively higher levels reported in neural tissues. At the cellular level, SBF2 is especially relevant in Schwann cells, where it interacts with MTMR2 to regulate membrane trafficking and myelin homeostasis. This tissue distribution aligns with the clinical manifestations of SBF2-related disorders, particularly peripheral demyelinating neuropathy (40). The majority of reported *SBF2* variants associated with CMT4B2 are truncating variants, including nonsense and frameshift mutations, supporting loss of function as the primary disease mechanism. The variant identified in our cases, p. Gly916Arg, is a missense change that may also result in loss of function through disruption of protein structure and stability. To the best of our knowledge, this is the first report of missense variant affecting *SBF2* as the plausible cause of CMT in the patients of Pakistani origin. Molecular modeling demonstrated an RMSD of 0.737 Å (Figure 2h) between the wild-type and mutant proteins, indicating noticeable conformational alterations. Furthermore, structural analyses predicted that the p. Gly916Arg substitution destabilizes the SBF2 protein (ΔΔG = −0.907 kcal/mol) and induces local perturbations that could compromise its functional integrity. In addition, the identified variant has an alpha missense score of 0.9993 (Strong pathogenic prediction) (41). Collectively, these findings support a deleterious effect of the variant and suggest a potential loss-of-function mechanism consistent with the established disease etiology of CMT4B2. However, these conclusions are based on *in silico* predictions, and additional functional studies will be required to experimentally validate the pathogenic impact of the variant.

Furthermore, all the five variants were not present in homozygous form in public database gnomAD. The mutated residues were found to be highly conserved across multiple species. When thoroughly examined after genetic analysis, affected individuals from all five families exhibited characteristic clinical features of CMT, including peripheral neuropathy, muscle atrophy, foot deformities (Figure 1C), distal sensory loss, and clawed hands. The patients also manifested Pes cavus and atrophy of the muscles (Figure 1C). The disease was also progressive in nature, because of which they become wheelchair dependent at the end of first decade of their life.

In summary, we identified four homozygous missense variants in *GDAP1* and one homozygous missense variant in *SBF2* in five independent consanguineous Pakistani families. Our combined findings not only expand the genetic spectrum of both *GDAP1* and SBF2 mutations causing CMT from a population that is largely uncharacterized due to lack of genomic facilities but also provide mechanistic insight into its pathogenicity using bioinformatics analysis. Furthermore, our study also highlighted the diagnostic importance of WES in identification of clinically and genetically heterogeneous neurological disorders. A limitation of this study is the absence of experimental functional validation of the novel variants reported in this study. Although segregation data, clinical findings, and *in silico* analyses support its pathogenicity, further functional studies are required to confirm its biological impact and disease mechanism.

## Data Availability

The original contributions presented in the study are included in the article/Supplementary material, further enquiries can be directed to the corresponding authors.

## Footnotes

1
http://biosig.unimelb.edu.au/mcsm/stability

## Supplementary material

SUPPLEMENTARY FIGURE 1 3D modeling of *GDAP1* and *SBF2* protein. **(A)** Represents the Alfa fold generated model of *GDAP1* protein. **(B)** Validation of *GDAP1* model by Ramachandran plot. **(C)** Structural validation of *GDAP1* protein by ProSA-web. **(D)** Represents the Alfa fold generated model of *SBF2* protein. **(E,F)** validation of *SBF2* protein model by Ramachandran and ProSA-web.

SUPPLEMENTARY FIGURE 2 Mutants modeling and superimposition. **(A)** Represents the Alfa fold generated model of GDAP1 protein. **(B)** Represents the Met1Val mutant model of GDAP1. **(C)** Showing deviation of Met1Val mutant as RMSD.
